# IoMT-Based Automated Detection and Classification of Leukemia Using Deep Learning

**DOI:** 10.1155/2020/6648574

**Published:** 2020-12-03

**Authors:** Nighat Bibi, Misba Sikandar, Ikram Ud Din, Ahmad Almogren, Sikandar Ali

**Affiliations:** ^1^Department of Information Technology, TheUniversity of Haripur, Haripur 22620, Pakistan; ^2^Department of Computer Science, College of Computer and Information Sciences, King Saud University, Riyadh 11633, Saudi Arabia; ^3^Beijing Key Laboratory of Petroleum Data Mining, China University of Petroleum-Beijing, Beijing 102249, China

## Abstract

For the last few years, computer-aided diagnosis (CAD) has been increasing rapidly. Numerous machine learning algorithms have been developed to identify different diseases, e.g., leukemia. Leukemia is a white blood cells- (WBC-) related illness affecting the bone marrow and/or blood. A quick, safe, and accurate early-stage diagnosis of leukemia plays a key role in curing and saving patients' lives. Based on developments, leukemia consists of two primary forms, i.e., acute and chronic leukemia. Each form can be subcategorized as myeloid and lymphoid. There are, therefore, four leukemia subtypes. Various approaches have been developed to identify leukemia with respect to its subtypes. However, in terms of effectiveness, learning process, and performance, these methods require improvements. This study provides an Internet of Medical Things- (IoMT-) based framework to enhance and provide a quick and safe identification of leukemia. In the proposed IoMT system, with the help of cloud computing, clinical gadgets are linked to network resources. The system allows real-time coordination for testing, diagnosis, and treatment of leukemia among patients and healthcare professionals, which may save both time and efforts of patients and clinicians. Moreover, the presented framework is also helpful for resolving the problems of patients with critical condition in pandemics such as COVID-19. The methods used for the identification of leukemia subtypes in the suggested framework are Dense Convolutional Neural Network (DenseNet-121) and Residual Convolutional Neural Network (ResNet-34). Two publicly available datasets for leukemia, i.e., ALL-IDB and ASH image bank, are used in this study. The results demonstrated that the suggested models supersede the other well-known machine learning algorithms used for healthy-versus-leukemia-subtypes identification.

## 1. Introduction

Internet of Things (IoT) is deployed in several areas, such as vehicular communications [1, 2], smart cites [3], cloud computing [4, 5], smart ecosystems [6, 7], smart campus [8], mobile communications [9], smart agriculture [10], and Healthcare or Internet of Medical Things (IoMT) [11]. However, a large section of the research community is attracted by IoMT or simply Healthcare IoT. IoMT [12, 13] is indeed a set of WiFi smart medical gadgets and smart applications connected to IT health systems through computer networks [14–16]. Smart medical devices are equipped with sensors or other computing resources and are exclusively intended for healthcare in the body at home, clinic, hospital, and community [17, 18]. These smart devices are linked to the cloud platforms to analyze collected data for further processing [19]. The IoMT technology includes virtual care for patients with long-term illnesses, portable mHealth devices for patients, monitoring of patients' medication, tracking the location of hospitalized patients [20, 21], and the ability to provide information to caregivers [22, 23]. The IoMT technology saves time and efforts of patients and doctors [11]. Connecting patients to their doctors and enabling the transfer of medical data over a secure network reduce the burden on health systems [24]. A rapid increase in the development and use of IoMT opens the door to deploying such frameworks that can fastly, securely, and accurately examine the patients' health and diagnose and cure different diseases remotely [25]. IoMT-based frameworks are abundant, particularly for those diseases which are more crucial with respect to patients' life, such as leukemia.

### 1.1. Leukemia

Leukemia is a disease related to white blood cells (WBC). Platelets, red blood cells (RBC), and WBC are various components of blood. Platelets help to clot and control bleeding. RBC known as erythrocytes are responsible for the transfer of oxygen through lungs to the body tissues. While WBC, also known as leukocytes, are responsible for fighting against diseases and infections. Leukemia refers to the production of large numbers of immature WBC. It is a type of cancer that affects the bone marrow and blood while destroying the immune system of a human body. Two main categories of leukemia based on progress are acute and chronic leukemia. Infected WBC grow rapidly in acute leukemia and do not perform in a normal way, whereas in chronic leukemia, WBC can act normally and grow less quickly. However, this can be severe since it may not be distinguished easily from the normal WBC. In addition, two types of acute and chronic leukemia are lymphoid and myeloid leukemia based on the size and shape of WBC, where both of them can be further divided into two subtypes each, i.e., acute lymphocytic leukemia (ALL), chronic lymphocytic leukemia (CLL), acute myeloid leukemia (AML), and chronic myeloid leukemia (CML).

#### 1.1.1. Acute Lymphocytic Leukemia

ALL is mostly seen in children, which is a WBC cancer caused by the constant multiplication and overproduction of immature WBC in the bone marrow. The symptoms of ALL are quite similar to flu and other common diseases, such as exhaustion, weakness, and pain in joints and bones, making it very difficult to diagnose this disease. Three types of ALL are classified as L1, L2, and L3 [26].

#### 1.1.2. Acute Myeloid Leukemia

The most common type of acute leukemia is AML, which happens when the bone marrow starts producing blasts and immature WBC. It may also create RBC and platelets that are abnormal. The common symptoms of early-stage AML may be similar to those of influenza or other common illnesses. Based on the types of blood cell affected, signs and symptoms may vary. The signs of AML are fever, bone pain, tiredness and fatigue, shortness of breath, pale skin, frequent infections, easy bruising, and abnormal bleeding, such as frequent nosebleeds and gum bleeding. AML has eight different subtypes that differentiate it from the other types of leukemia [27].

#### 1.1.3. Chronic Lymphoblastic Leukemia

CLL is a hematological sickness that gets worse slowly. It is commonly observed in adults and is very uncommon in children. The symptoms of CLL include loss in weight, fever, night sweats, and periodic infections.

#### 1.1.4. Chronic Myeloid Leukemia

**Figure 1:**
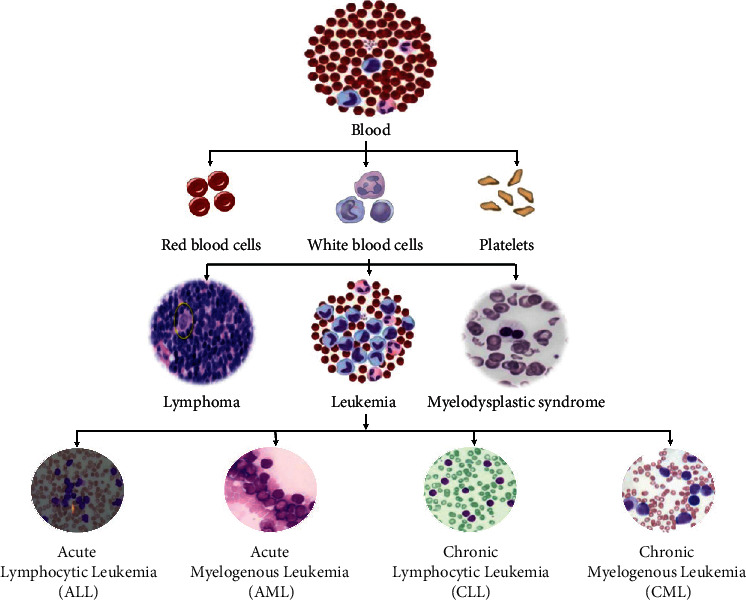
Blood and leukemia types.

CML, also known as chronic myelogenous leukemia, is a form of slow growing leukemia, but it can develop into acute leukemia, which is fast growing and difficult to treat. This may be viewed in three stages, i.e., chronic, accelerated, and blast stages. In the chronic stage, the leukemia is inside the strongest situation and grows slowly. Within the second stage, the blood cells are immature, usually referred to as extended stage. The third stage is the blast stage, which is also known as the acute stage or blast transformation stage. The pictorial representation of blood structure and leukemia types is shown in Figure 1.

It is necessary for hematologists to identify the existence of leukemia along with its particular form in order to avoid medical risks and to determine the correct leukemia treatment. A crucial and time-consuming step is the identification of leukemia through optical blood smears examination monitored by a specialist. To solve such problems, many CAD methodologies for quantitative analysis of the peripheral blood samples have been developed using machine learning and deep learning methods. However, these approaches have some drawbacks and need improvements in terms of accuracy, learning process, and efficiency.

**Figure 2:**
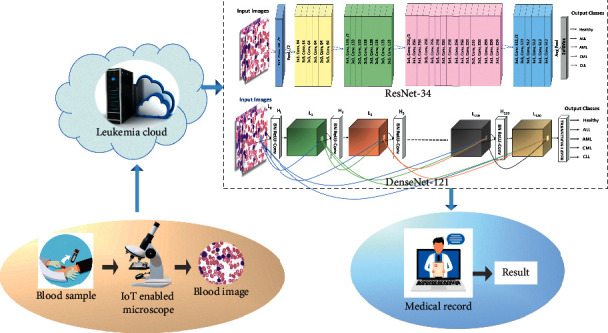
Proposed framework for automated leukemia diagnosis.

Thus, to tackle these drawbacks and by keeping in view the vitality of healthcare, an IoMT-based framework for the automatic identification of leukemia subtypes is proposed in this study. In the proposed framework, IoT-enabled microscope uploads the blood smear images to the leukemia cloud. The leukemia with respect to its type(s) is diagnosed by using the deep learning models, i.e., ResNet-34 [28] or DenseNet-121 [29]. Deep learning is a branch of machine learning used to solve a variety of problems and describes the abstract concepts through different layers of data processing to discover better learning algorithms and representations that are less dependent on feature engineering [30]. The high prediction power of deep learning algorithms and surprising ability of features extraction extend their use to a wide range of research areas. Therefore, in this study, deep transfer learning-based methods are proposed to identify microscopic blood images as healthy, ALL, AML, CLL, and CML. The detail of the models is given in Section 3. The diagnostic results of leukemia with respect to its types predicted by the suggested models can be shown on the clinician's computer and accordingly, medical care may be offered to leukemia patients. The proposed framework is demonstrated in Figure 2.

The suggested framework is also helpful for patients and doctors in pandemics such as coronavirus disease 2019 (COVID-19). Due to the spread of COVID-19, most of the countries imposed sudden lockdown in major cities; as a result, almost ten billion citizens were quarantined. During this pandemic, people put their focus on accumulating more necessary care. However, some patients with chronic diseases leave their homes for medical help opening a gap in quarantine measures, which is a threat to disease control. Hence, if the proposed framework is applied to the current crisis, it would provide a medical platform that may help patients to receive adequate medical care at homes. The proposed approach is fast and accurate in addition to reducing the need for an expert oncologist to diagnose leukemia or any of its subtypes.

We delineate our experiments in the following sections. The related work is presented in Section 2.  Section 3 elaborates the data exploration, augmentation, and the proposed models.  Section 4 describes the experimental results, while Section 5 concludes the conducted study and presents future research directions.

## 2. Literature Survey

For the computer-aided diagnosis (CAD) of leukemia, various studies have been conducted by the IoMT research community. These studies present different machine and deep learning algorithms for the identification of leukemia. In [31], the classification and detection of WBC cancer and some of its subtypes are done by using Random Forest with 94.3% accuracy. In [32], the proposed model detected ALL using KNN and Naive Bayes Classifier with 92.8% accuracy. The classifier is tested on 60 sample images. In [33], a novel Principal Component Analysis (PCA) based on ABC-BPNN scheme is suggested for the classification of leukemia cells and attained 98.72% average accuracy with reduction in the computation time. In [34], the ALL is identified. Firstly, leukemia images are segmented by BSA-based clustering. Secondly, Jaya technique is applied in integration with some standard classification methods such as Naive Bayes, K-nearest neighbor, linear discriminant analysis, support vector machine (SVM), ensemble random under-sampling boost, and decision tree. However, Jaya with SVM and Jaya with decision tree gave better accuracy.

The Linear Discriminant Analysis- (LDA-) based PCA model for diagnosing leukemia by utilizing Discrete Orthogonal Stockwell Transform (DOST) method to extract the features of microscopic images is presented in [35]. In [36], for feature extraction, three pre-trained CNN architectures are applied. However, for the leukemia classification on the hybrid database, SVM without segmentation is used. In [37], for the identification of acute leukemia from microscopic images initially to extract features, the images were segmented by using unsharp masking sub-imaging bounding box and L × a × b color Fuzzy C-Means Clustering after applying the Nearest Neighbor. Then, to identify ALL, the extracted features were passed to SVM, which attained 95% accuracy. In [38], a robust feature extraction and selection technique for the identification of lymphocytes versus ALL is proposed. The classification is done by KNN using Euclidean Distance with 92.5% accuracy. In [39], the classification is done using CNN, where only ALL vs healthy samples are classified with 88.25% accuracy and leukemia subtypes are classified with 81.74% accuracy.

In [35, 40–43], ALL-IDB dataset is used to detect ALL. In [40], K-medoids is presented with 98.60% accuracy. In [35], DOST, PCA, and LDA are proposed with 99.66% accuracy. In [41], Generative Adversarial Optimization- (GAO-) based method is investigated with 93.84% accuracy. In [42], Genetic Algorithm (GA) and Artificial Neural Network (ANN) are presented with 97.07% accuracy. In [43], Chronological Sine Cosine Algorithm (SCA) is tested, which achieved 98.70% accuracy. In [44], ASH image bank and ALL-IDB1 datasets are utilized for classifying lymphoblast cells. Convnet is investigated and achieved 81.74% accuracy. In [36], heterogeneous database ALL-IDB1 and ALL-IDB2 datasets are used. The diagnosis of leukemia (pathological or non-pathological) is done using pre-trained CNN with SVM, which attained 99% accuracy. In [45], the ALL-IDB1 dataset is employed where the diagnosis of leukemia (normal vs abnormal) is performed by CNN and achieved 96.60% accuracy.

In [46], the ALL-IDB1 and ALL-IDB2 datasets are employed for the ALL detection. However, the scheme used is SVM, which attained 89.81% accuracy. In [47], the ALL-IDB2 dataset is used for the identification of ALL where the method investigated is customized KNN with 96.25% accuracy. In [48], by utilizing ALL-IDB1 dataset, ALL is classified on the basis of cell energy features by employing SVM, which attained 94.00% accuracy. In [49], by utilizing ASH image bank dataset, firstly FAB ALL subtypes are identified by applying GA with multilayer perceptron kernel (MLP) function and acquired 97.1% accuracy. Secondly, FAB AML subtypes are identified by employing genetic phenomena with Gaussian radial basis kernel function and achieved 98.5% accuracy. Finally, healthy, ALL, and AML are identified by using a GA with Gaussian Radial Basis kernel and achieved 99.50% accuracy.

It is exhibited from the literature that almost all previous approaches identified leukemia with respect to healthy, AML or ALL types. However, these approaches did not address the problem of identifying leukemia with respect to all its subtypes, i.e., ALL, AML, CLL, and CML. Therefore, in this study, the deep CNN-based approaches are presented to classify leukemia in terms of all its types.

The proposed work is an advancement of the study conducted in [39], where the authors have done the classification of ALL vs. healthy using a simple CNN architecture. However, our proposed work focuses on the classification of four subtypes of leukemia, i.e., ALL, AML, CLL, and CML, using advanced CNN architectures—Residual Network-34 (ResNet-34) [28] and Dense Convolutional Network-121 (DenseNet-121) [29]. Hence, it is elaborated in the result section that the proposed deep CNNs, i.e., DenseNet-121 and ResNet-34, outperform the existing schemes with 99.91% and 99.96% accuracy, respectively. Furthermore, for investigating the proposed methods, ALL-IDB and ASH image bank datasets are utilized.

## 3. Experimental Setup

**Figure 3:**
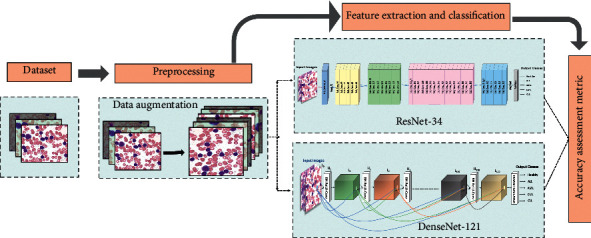
Proposed methodology.

This section delineates the experimental setup for conducting the study. Firstly, the dataset retrieval is described followed by data augmentation, while in the end, the deep learning models used to classify the subtypes of leukemia are explained. The proposed methodology is presented in Figure 3.

### 3.1. Leukemia Database Description

The dataset is collected from two different sources: ASH image bank [50] and ALL-IDB [51, 52]. The ASH image bank is freely accessible on the Internet and contains a complete bank of images on a variety of hematological topics. In this article, all accessible annotated cell images with leukemia of blood are selected including any one of the four subtypes.

**Figure 4:**
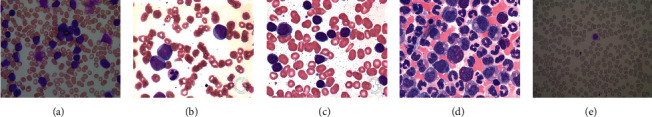
Leukemia subtype images. (a) Acute lymphocytic leukemia (ALL). (b) Acute myelogenous leukemia (AML). (c) Chronic lymphocytic leukemia (CLL). (d) Chronic myelogenous leukemia (CML). (e) Healthy.

The ALL-IDB dataset provides annotated microscopic images of blood cells that were developed for segmentation, evaluation, and classification. ALL-IDB contains only healthy and ALL types of leukemia samples. The remaining mentioned leukemia subtypes are not available in the ALL-IDB dataset. The ALL-IDB is considered more reliable because experienced oncologists provided the ALL classification for each image in the dataset. Some samples of images from datasets are shown in Figure 4.

### 3.2. Reducing Overfitting

Data augmentation methods are commonly used for maximizing the size of datasets and reducing overfitting, especially in the deep learning models. Various image transformation schemes, such as rotation, flipping, and shifting, have been used to obtain distinct images from the original image. They would have more generalization capabilities if machine learning and deep learning models were trained with the original images along with their sub-versions. In different image segmentation studies with CNNs, numerous methods of information replication have reduced the model error rate by giving better speculation. Here, a better assortment of microscopic blood cell images is provided by the retrieved datasets, but both datasets contain samples of limited numbers of leukemia subtypes. As the sample datasets are very limited for deep learning techniques, it can lead to overfitting. Thus, image transformation or data augmentation techniques are used to maximize the dataset; i.e., rotation, height shift, width shift, horizontal flip, zoom, and shearing, are applied. The sample size after applying the methods of image transformation is increased in both datasets. Exact number of samples per leukemia subtype before and after augmentation is shown in Table 1. After applying the data augmentation, the sample size reached 3277 and 2359 in the ASH image bank and ALL-IDB datasets, respectively.

### 3.3. Deep Learning Models

After performing augmentation on the dataset, it is passed on to CNNs—the deep learning models. Instead of using traditional machine learning techniques which are provided with hand-crafted features to learn and require time and effort, in this study, CNNs are utilized. CNNs have the ability to learn automatically from raw data. A typical CNN begins with convolutional and pooling layers and ends with a fully connected layer. New models can be created from a CNN by assembling some convolutional, pooling, and dense layers. By arranging some layers of CNNs architectures, new light CNN models have been developed such as VggNet [53] and AlexNet [54]. However, advanced CNN models, such as ResNet [28] and DenseNet [29], are deeper and more complex having the ability to learn better, as elaborated in the result section. Therefore, in the presented work, ResNet-34 and DenseNet-121 models are used in a supervised learning mode to identify and categorize leukemia with respect to its types. The details of each model are described in the following subsections.

#### 3.3.1. ResNet-34

ResNet-34 is a pre-trained 34-layer model. The performance of deep neural networks depends on architecture and dataset. A deep network of CNNs and large dataset produce better performance. However, the performance deteriorates after a certain depth when the network gets deeper. The reason of this problem is the vanishing gradient. The ResNet solves this problem as gradients flow from starting layers to the final ones by skipping some layers. Mathematically, the layers of the ResNet model can be calculated according to

(1) $$\begin{array}{c} Y=f\left(x\right)+id\left(x\right)=f\left(x\right)+x. \end{array}$$

**Figure 5:**
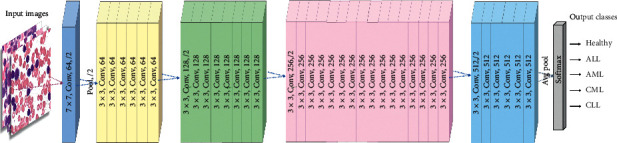
General architecture of ResNet-34 (adapted from [28]).

By skipping the connections between layers, the gradient can easily flow and the training of the layers becomes faster. ResNet-34 consists of a total of 34 layers wherein one is convolutional and pooling layer in addition to four other layers with the same pattern. Each layer is convolved with 3 × 3 convolution with a feature map of sizes 64, 128, 256, and 512, respectively [28]. The general architecture of RestNet-34 is presented in Figure 5.

#### 3.3.2. DenseNet-121

**Figure 6:**
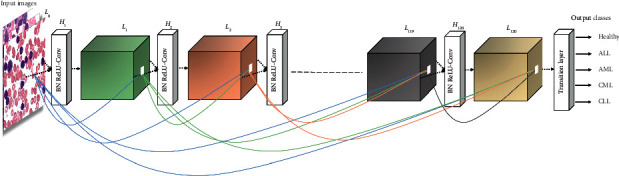
General architecture of DenseNet-121 (adapted from [29]).

Dense convolutional networks or DenseNet achieved the best classification results on CIFAR-10 and ImageNet datasets [29]. Dense connections are used in the DenseNet architecture, such as ResNet architecture. DenseNet-121 consists of 121 layers. In DenseNet architecture, each layer is connected to all subsequent layers. Thus, each layer receives important features learned by any preceding layers of the network that makes training of the network more efficient [55]. The DenseNet architecture uses fewer parameters than ResNet for the training of the network. Small datasets make the model overfit, while the dense connection solves that overfitting problem [29]. A significant part of DenseNet is a dense block, which is used for enhancing the information flow between layers. It consists of BN, ReLU, and 3×3 conv. The particular formula for the dense block is provided in

(2) $$\begin{array}{c} L_{l}=H_{l}\left(\left[L_{0},L_{1},\ldots ,L_{l-1}\right]\right), \end{array}$$

where (0, 1,…, *l*-1) represents the layers of DenseNet-121 and [*L*_0_, *L*_1_,…, *L*_*l*−1_] is the concatenation of feature map obtained from the layers of DenseNet-121. The composite function of BN, ReLU, and 3 × 3 conv. operations on the *l*^th^ layer is presented by *Hl*(.) [29]. The DenseNet architecture is presented in Figure 6.

Both pre-trained models are implemented with *Python* using open source fastai [56] and the deep learning library, which makes the implementation of the models simpler. All the experiments are performed on Google Colab [57].

## 4. Experimental Results

In order to evaluate the proposed models, the performance measures used are precision, recall, F1 score, and accuracy. The mathematical description of each of these parameters is given in Table 2. Here, TP is a true positive rate and refers to positive class determined as positive; FP is false positive rate and refers to negative class determined as positive; TN is true negative rate and refers to negative class determined as negative; and FN is false negative rate and refers to positive class determined as negative [58]. However, Recall is the accuracy of prediction for the known leukemia subtype class. Accuracy is the prediction for the known leukemia and non-leukemia subtype classes [59]. Precision is the ratio of correct positive predicted leukemia subtype classes to the predicted positive leukemia subtype classes, while F1 score is the harmonic mean of precision and recall [23].

**Figure 7:**
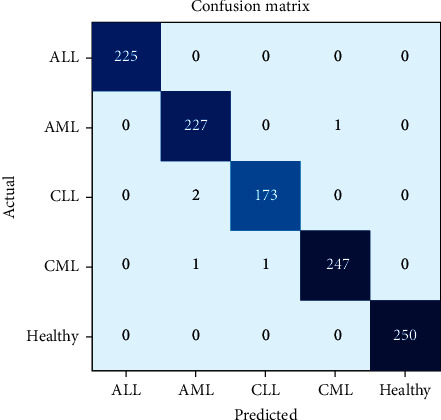
Confusion matrix of ResNet-34 for leukemia subtype classification.

**Figure 8:**
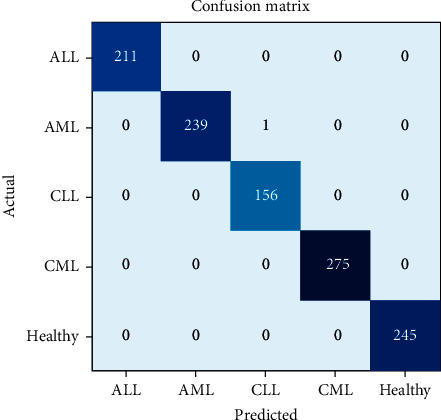
Confusion matrix of DenseNet-121 for leukemia subtype classification.

To show the efficiency of the utilized methods, various parameters such as precision, recall, F1 score, and accuracy are used. Figures 7 and 8 show the confusion matrix for the classification of subtypes of leukemia using ResNet-34 and DenseNet-121, respectively. It is evident from Figures 7 and 8 that the proposed models predicate very well. Furthermore, Tables 3 and 4 present the accuracy, precision, recall, and F1 score for each type of leukemia on the benchmark dataset, ALL-IDB and ASH image bank.

It is exhibited from Tables 3 and 4 that the ResNet-34 and DenseNet-121 prediction accuracy for ALL and healthy cases is 100%, while precision, recall, and F1 score are also 100%, i.e., 1.0. The prediction accuracy of ResNet-34 for AML is 99.65%, precision is 1.0%, recall is 0.99%, and F1 score is also 0.99%. In case of CLL, the prediction accuracy of ResNet-34 is 99.73%. However, precision, recall, and F1 score are 0.99%. For CML, the prediction accuracy of ResNet-34 is 99.73%, precision is 0.99%, recall is 1.0%, and F1 score is 0.99%, whereas in case of DenseNet-121, the prediction accuracy for AML is 99.91%. However, precision, recall, and F1 score are 1.0%. In case of CLL, the prediction accuracy of DenseNet-121 is 99.91%, precision is 1.0%, recall is 0.99%, and F1 score is 1.0%. For CML, the DenseNet-121 prediction accuracy, precision, recall, and F1 score are 100%.

**Figure 9:**
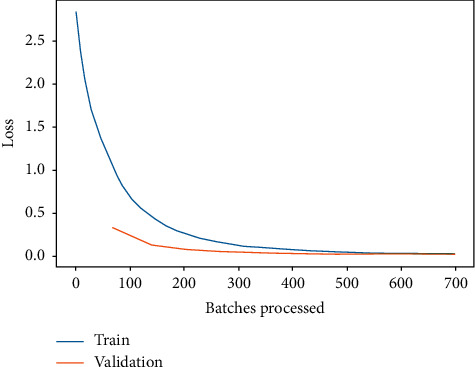
Training and validation loss of ResNet-34.

**Figure 10:**
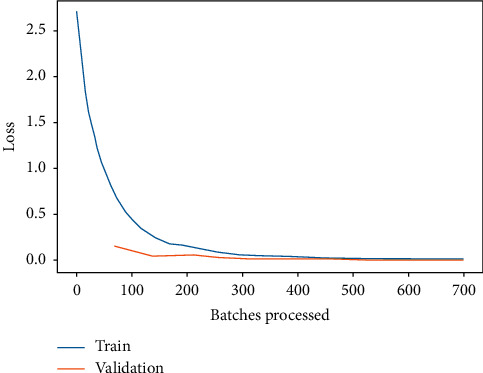
Training and validation loss of DenseNet-121.

**Figure 11:**
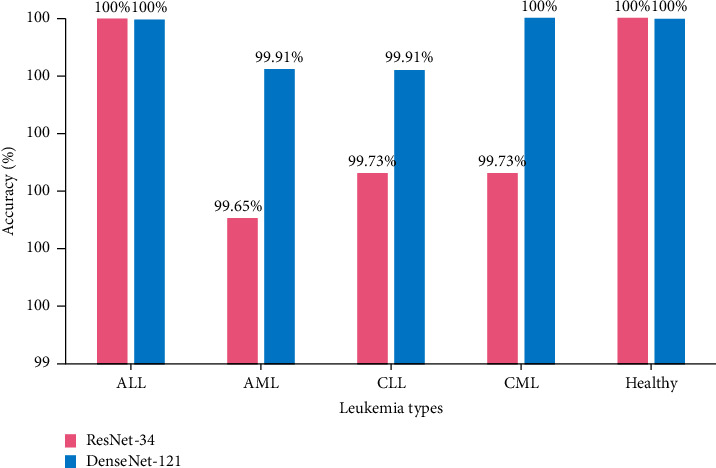
Accuracy comparison of the ResNet-34 and DenseNet-121 for leukemia subtype classification.

Moreover, the training and validation loss for ResNet-34 and DenseNet-121 are shown in Figures 9 and 10, respectively. It is depicted from Figures 9 and 10 that the training and validation loss of ResNet-34 and DenseNet-121 are nearly 0, which means that the accuracy is also near 100%. However, in case of DenseNet-121, the training and validation loss are nearer 0 as compared to ResNet-34. Hence, it is concluded from the results that the prediction performance of ResNet-34 and DenseNet-121 is better for identifying the leukemia subtypes. However, DenseNet-121 seems to supersede ResNet-34. The DenseNet-121 training and validation loss are near 0 when compared to ResNet-34, while for some subtypes identification, i.e., AML, CLL, and CML, DenseNet-121 outperforms ResNet-34. The performance comparison of ResNet-34 and DenseNet-121 with respect to leukemia subtypes identification is shown in Figure 11.

**Figure 12:**
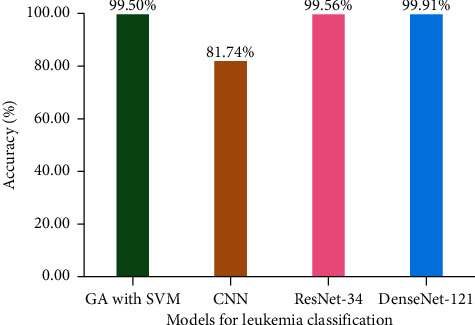
Comparison of the studies on automated detection of subtypes of leukemia.

To show their efficiency, a comparison of the proposed models is done with previous approaches, i.e., GA with SVM [49] and CNN [39]. GA with SVM performs the identification of ALL, AML, and healthy samples, while CNN performs the leukemia subtype identification. Nevertheless, it is elucidated in Figure 12 that the utilized models, i.e., ResNet-34 and DenseNet-121, outperform the existing schemes, such as GA with SVM and CNN. It is exhibited from Figure 12 that GA with SVM has 99.50%, CNN has 81.74%, ResNet-34 has 99.56%, and DenseNet-121 has 99.91% accuracy, respectively. Thus, DenseNet-121 supersedes the other approaches. Previously, numerous machine learning techniques were using the same datasets, i.e., ALL-IDB and ASH image bank, for the detection of leukemia and its subtypes. In those studies, the feature extraction and classification methods were used, which require time and effort. However, the proposed models do not need hand-crafted feature set for making predictions and save time and efforts. A thorough comparison of the proposed models with the previous approaches with respect to accuracy is presented in Table 5. It is depicted from Table 5 that the proposed models outperform the previous approaches with average accuracy of 99.56% and 99.91% for ResNet-34 and DenseNet-121, respectively.

## 5. Conclusion and Research Directions

In this study, an IoMT-based framework is proposed for the leukemia subtypes detection. In the proposed framework, an IoT-enabled microscope uploads the blood smear images to the leukemia cloud. The leukemia is diagnosed by using the ResNet-34 or DenseNet-121 models. It is observed that the diagnosing power of ResNet-34 and DenseNet-121 supersedes all the previous approaches. By using data augmentation techniques, ResNet-34 and DenseNet-121 both process large numbers of image patterns. After diagnosis, the result is sent to the doctor's computer where s/he provides medical care on the basis of test report through the IoMT framework. Furthermore, the proposed framework facilitates the patients in pandemics such as COVID-19.

In the future, the dataset can be extended by adding new samples of blood images and utilizing new augmentation techniques to achieve better performances. Furthermore, the proposed IoMT-based framework can be equipped with the functionality of diagnosing the subcategories of each leukemia type. Moreover, the proposed models can also be used to find other abnormalities in the blood.

## Figures and Tables

**Table 1:** Distribution of leukemia subtypes before and after augmentation.

Leukemia type	Dataset	Before augmentation	After augmentation
ALL	ALL-IDB	181	1079
AML	ASH image bank	55	1194
CLL	ASH image bank	38	840
CML	ASH image bank	57	1243
Healthy	ALL-IDB	187	1280

**Table 2:** Performance measures mathematical description.

Measure	Derivations
Accuracy	ACC = (TP + TN)/(P + N)
Precision	PPV = TP/(TP + FP)
Recall	TPR = TP/(TP + FN)
F1 score	F1 = 2TP/(2TP + FP + FN)

**Table 3:** Performance of the ResNet-34 model for leukemia subtype classification.

Leukemia type	Accuracy	Precision	Recall	F1 score
ALL	100	1.0	1.0	1.0
AML	99.65	1.0	0.99	0.99
CLL	99.73	0.99	0.99	0.99
CML	99.73	0.99	1.0	0.99
Healthy	100	1.0	1.0	1.0

**Table 4:** Performance of the DenseNet-121 model for leukemia subtype classification.

Leukemia type	Accuracy	Precision	Recall	F1 score
ALL	100	1.0	1.0	1.0
AML	99.91	1.0	1.0	1.0
CLL	99.91	1.0	0.99	1.0
CML	100	1.0	1.0	1.0
Healthy	100	1.0	1.0	1.0

**Table 5:** A comparison of the proposed models with the previous approaches for automated detection of leukemia and its subtypes using the same datasets with respect to average accuracy.

Reference	Dataset	Classification	Classifier	Accuracy (%)
Ahmed et al. [39]	ALL-IDB	Leukemia vs healthy	CNN	88.25
			Naive Bayes	69.69
			Decision tree	62.94
			KNN	58.57
			SVM	50.09
	ALL-IDB, ASH image bank	Leukemia subtypes classification	CNN	81.74
			Naive Bayes	52.68
			Decision tree	45.92
			KNN	43.51
			SVM	20.84
Shafique et al. [26]	ALL-IDB	Acute lymphoblastic leukemia detection	AlexNet	99.50
		Subtypes of acute lymphoblastic leukemia	AlexNet	96.06
Jothi et al. [34]	ALL-IDB	Acute lymphoblastic leukemia detection	Jaya, SVM	99.00
			Jaya, decision tree	98.00
Acharya et al. [40]	ALL-IDB	White blood cells	K-medoids algorithm	98.60
Mishra et al. [35]	ALL-IDB1	Acute lymphoblastic leukemia detection	DOST, PCA, LDA	99.66
Tuba et al. [41]	ALL-IDB2	Acute lymphoblastic leukemia detection	GAO-based methods	93.84
Al-jaboriy et al. [42]	ALL-IDB1	Acute lymphoblastic leukemia detection	GA and ANN	97.07
Jha et al. [43]	ALL-IDB2	Acute lymphoblastic leukemia detection	SCA-based deep CNN	98.70
Pansombut et al. [44]	ASH image bank, ALL-IDB1	Lymphoblast cells	CNN-based convnet	81.74
Vogado et al. [36]	Heterogeneous database ALL-IDB1, ALL-IDB2	Diagnose leukemia (pathological or not)	Pre-trained CNN with SVM	99
Thanh et al. [45]	ALL-IDB1	Diagnose leukemia (normal vs abnormal)	CNN	96.60
Moshavash et al. [46]	ALL-IDB1, ALL-IDB2, Dr. Juan Bruno Zayas Alfonso Hospital, Santiago de Cuba	Acute lymphoblastic leukemia detection	Two ensemble classifiers with SVM	89.81
Umamaheswari et al. [47]	ALL-IDB2	Acute lymphoblastic leukemia	Customized KNN	96.25
Agaian et al. [48]	ALL-IDB1	Acute lymphoblastic leukemia	Cell energy feature with SVM	94.00
Rawat et al. [49]	ASH image bank	ALL	GA with SVM	97.10
		AML		98.50
		Healthy, AML,ALL		99.50
Proposed work	ALL-IDB, ASH image bank	Healthy, ALL, AML, CLL and CML	ResNet-34	99.56
			DenseNet-121	99.91

## Data Availability

The following two datasets have been used in the research: ASH image bank and ALL-IDB publicly available datasets, which have been cited in the paper at proper positions.
